# Machine-learned models using hematological inflammation markers in the prediction of short-term acute coronary syndrome outcomes

**DOI:** 10.1186/s12967-018-1702-5

**Published:** 2018-12-03

**Authors:** Konrad Pieszko, Jarosław Hiczkiewicz, Paweł Budzianowski, Janusz Rzeźniczak, Jan Budzianowski, Jerzy Błaszczyński, Roman Słowiński, Paweł Burchardt

**Affiliations:** 1 Faculty of Medicine and Health Sciences, University of Zielona Gora, Zielona Gora, Poland; 2 Department of Cardiology, Nowa Sol Multidisciplinary Hospital, Szpital w Nowej Soli, Oddział Kardiologii, 67-100 Nowa Sol, Poland; 3 Biology of Lipid Disorders Department, Poznan University of Medical Sciences, Poznan, Poland; 4 Department of Cardiology, J Strus Hospital, Poznan, Poland; 5 Laboratory of Intelligent Decision Support Systems, Poznań University of Technology, Poznan, Poland; 6 Department of Engineering, University of Cambridge, Cambridge, UK

**Keywords:** Acute coronary syndrome, Machine learning, Risk assessment, Biomarkers, Inflammation, Outcomes research

## Abstract

**Background:**

Increased systemic and local inflammation play a vital role in the pathophysiology of acute coronary syndrome. This study aimed to assess the usefulness of selected machine learning methods and hematological markers of inflammation in predicting short-term outcomes of acute coronary syndrome (ACS).

**Methods:**

We analyzed the predictive importance of laboratory and clinical features in 6769 hospitalizations of patients with ACS. Two binary classifications were considered: significant coronary lesion (SCL) or lack of SCL, and in-hospital death or survival. SCL was observed in 73% of patients. In-hospital mortality was observed in 1.4% of patients and it was higher in the case of patients with SCL. Ensembles of decision trees and decision rule models were trained to predict these classifications.

**Results:**

The best performing model for in-hospital mortality was based on the dominance-based rough set approach and the full set of laboratory as well as clinical features. This model achieved 81 ± 2.4% sensitivity and 81.1 ± 0.5% specificity in the detection of in-hospital mortality. The models trained for SCL performed considerably worse. The best performing model for detecting SCL achieved 56.9 ± 0.2% sensitivity and 66.9 ± 0.2% specificity. Dominance rough set approach classifier operating on the full set of clinical and laboratory features identifies presence or absence of diabetes, systolic and diastolic blood pressure and prothrombin time as having the highest confirmation measures (best predictive value) in the detection of in-hospital mortality. When we used the limited set of variables, neutrophil count, age, systolic and diastolic pressure and heart rate (taken at admission) achieved the high feature importance scores (provided by the gradient boosted trees classifier) as well as the positive confirmation measures (provided by the dominance-based rough set approach classifier).

**Conclusions:**

Machine learned models can rely on the association between the elevated inflammatory markers and the short-term ACS outcomes to provide accurate predictions. Moreover, such models can help assess the usefulness of laboratory and clinical features in predicting the in-hospital mortality of ACS patients.

## Background

Many studies have shown that increased systemic and local inflammation play a key role in the pathophysiology of ACS. Hematological and inflammatory markers may have a meaningful predictive value for ACS outcomes [1]. Hence, readily available and inexpensive markers such as neutrophil count, neutrophil to lymphocyte ratio (NLR), red cell distribution width (RDW), platelet to lymphocyte ratio (PLR), mean platelet volume (MPV), and platelet distribution width (PDW) have recently attracted more attention and encouraged further research. Indeed, these indices may provide information on ACS pathophysiology and may be useful in risk stratification and its optimal management [2, 3]. Also, many studies have pointed at their prognostic value in all-cause mortality, major cardiovascular events, stent thrombosis, arrhythmias, and myocardial perfusion disorders concerning acute myocardial infarction and unstable angina [4]. The most recent studies have indicated that combining these markers with the Global Registry of Acute Coronary Events (GRACE), SYNTAX, and Thrombolysis in Myocardial Infarction (TIMI) scores improves risk stratification and ACS patients’ diagnostics [5–9].

With the growing availability of medical data, machine learning methods offer a promising extension of classical statistical analysis [10]. In this study, we have used machine learning methods and investigated the usefulness of the hematological indices presented above in predicting SCL and in-hospital mortality. We also demonstrated that machine learning methods can be a valuable supplement to the traditional methods of inferential statistics.

## Methods

We analyzed the medical records of patients with ACS admitted to the local cardiology unit between January 2012 and June 2017. The analyzed group comprised of patients who had their diagnosis reevaluated and confirmed by a cardiologist according to ESC guidelines [11]. The data concerning the 6769 hospitalizations (5678 individual patients) was obtained retrospectively from electronic medical records.

Two sets of features were considered in this study: a full set and a simplified set. Table 1 presents the variables used in both sets. The full set included 53 nominal and numeric features. All the variables were obtained from electronic medical records directly. Some information including descriptions of electrocardiograms or elements of physical examination was stored in our records as an unstructured text. Although some studies on ACS outcomes also set out to investigate the possibility of using the features extracted from unstructured reports [12], we decided to include only the features that were saved in our records directly to avoid additional bias.

**Table 1:** Features used by XGboost and DRSA-BRE classifiers

XGboost	DRSA-BRA (simplified set of features)	DRSA-BRE (full set of features)
1. Diastolic blood pressure 2. Systolic blood pressure 3. Troponin elevation ratio 4. Age 5. Heart rate 6. Mean platelet volume 7. Neutrophil to lymphocyte ratio 8. Weight 9. Creatinine level 10. Eosinophil count 11. Red cell distribution width 12. Height 13. Hematocrit 14. Body mass index 15. Platelet count 16. HDL level 17. Fibrinogen level 18. Lymphocyte count 19. Platelet to lymphocyte ratio 20. LDL level		1. All features from simplified set AND 2. Diabetes status Impaired glucose tolerance Type 1 diabetes Type 2 diabetes 3. Smoking status Former smoker Smoker Non-smoker 4. Mean cell volume 5. Triglycerides level 6. Sodium level 7. Potassium level 8. TSH level 9. Total cholesterol level 10. Urea level 11. Monocyte count 12. Hemoglobin level 13. Sodium level 14. Aspartate aminotransferase level 15. Alanine aminotransferase level 16. History of pulmonary disease 17. Hypertension 18. History of previous stroke 19. Basophile count 20. History of renal failure 21. Activated partial thromboplastin time 22. History of heart failure 23. Previous coronary artery bypass grafting 24. Sex 25. History of myocardial infarction 26. History of coronary heart disease 27. Family history of coronary heart disease 28. History of percutaneous coronary interventions 29. History of peripheral artery disease

The simplified set consisted of 23 numerical features. This set was chosen on the basis of its potential application and the potential predictive value for ACS outcomes. We favored the features that did not require human interpretation or analysis. In this way, we tried to investigate the possibility of creating a classifier that could be built into medical records software and automatically identify the patients with a high risk of an unfavorable outcome.

The inclusion criteria for the study were as follows:

1. The patient was admitted to the cardiology department on an emergency basis.
2. The patient had a discharge diagnosis of ACS including STEMI, NSTE-ACS or unstable angina.
3. The patient had coronary angiography within 96 h of admission.
4. If the same patient was admitted multiple times in the analyzed period, each admission was recorded independently but the information about prior PCI, CABG or MI was retained.

Patients who were assessed to qualify for revascularization based on coronary angiogram and, therefore, underwent PCI or were referred to CABG were considered to have had significant coronary lesion (SCL) (n = 4943, 73% of cases), while patients who did not undergo revascularization were considered to have no sCAD (n = 1826, 27% of cases). Patients who did not consent for invasive management were excluded from the study.

In-hospital death was observed in 1.4% of cases (n = 97). Descriptive statistics were performed using the STATISTICA software. First, the normality of distribution was tested using the Shapiro–Wilk Test. The univariate two-tailed Mann–Whitney-U test and frequency tables were used to explore the differences between these two groups.

As a part of our study, we used machine learning methods and investigated their performance in predicting the presence of SCL and in-hospital mortality. However, we were not only interested in their predictive performance. The secondary aim of our study was to identify the extent to which the selected features affected the prediction accuracy. In particular, we wanted to investigate the predictive value of hematological indices and explore the possibility of creating a model based on them. That is why, the interpretability of the constructed classification model and its ability to identify significant features were of crucial relevance.

We considered three different classification algorithms: logistic regression, gradient boosted trees (XGBoost) and the Dominance-based Rough Set Balanced Rule Ensemble (DRSA-BRE). The logistic regression model was included in this study as a baseline classifier. Gradient boosted trees, by contrast, were used as a well-known and well-performing off-the-shelf classifier [13]. DRSA-BRE was explicitly included in the study due to the class imbalance in the dataset (i.e. the disproportion between the number of cases in classes) observed in both ACS problems. More precisely, in the DRSA-BRE undersampling neighborhood balanced bagging method [14] was applied to address the class imbalance problem. This type of classifier has recently been successfully applied to the Diabetic Retinopathy Assessment [15]. Additionally, to improve the predictive performance of XGBoost on the class-imbalanced problems, we undersampled the majority class in training sets.

When using logistic regression and XGBoost classifiers, the missing values were filled in with the mean values from all the observations in the test set. Moreover, both logistic regression and XGBoost were trained only on the simplified set of features. Both of these classifiers were not able to handle nominal values directly and thus we decided not to transform them. The DRSA-BRE classifier was trained on both the full and simplified sets of features. The missing values were handled directly in DRSA-BRE by the VC-DomLEM [16, 17] algorithm, which was used as a component classifier in the constructed bagging ensemble.

As explained above, one of the aims of our study was to assess the predictive importance of the analyzed sets of features on the short-term ACS outcomes. Our study showed that the XGBoost classifier provided the feature importance scores which reflected how valuable each feature was during the model construction. For the DRSA-BRE classifier the attribute relevance was evaluated by a confirmation measure (the degree to which the presence of an attribute in the hypothesis of a rule indicates accurate prediction). The higher the value of the confirmation measure the more important the attribute was [18, 19].

The model selection, optimization and fitting of the logistic regression and XGBoost models were performed using the scikit-learn [20] and XGboost [13] software packages. DRSA-BRE analysis was performed using the jRS library and jMAF software package [21] which are available for download at http://www.cs.put.poznan.pl/jblaszczynski/Site/jRS.html. The plots and visualizations were generated using the matplotlib [22] software package.

We focused our analysis on four performance metrics: sensitivity, specificity, G-mean and AUC. Sensitivity is defined as a ratio of the predicted genuine positive cases to all positive cases. Specificity is defined as a ratio of the predicted genuine negative cases to all negative cases. Receiver operating characteristics (ROC) curve analysis is a popular tool to analyze classifier performance. More precisely, classifier performance is reflected by the area under the ROC curve (so-called the AUC measure) [23].

Interestingly, however, some researchers have shown that AUC analysis has limitations. For example, in the case of highly skewed class distribution (i.e. class imbalanced problems) it may lead to an overoptimistic estimate of classifier performance [24]. That is why, we also verified simpler measures which are useful for the classifiers providing a purely deterministic prediction (see discussions on the applicability of ROC analysis in [25]). This measure is called G-mean and it is defined as a geometric mean of sensitivity and specificity [26].

## Results

The basic descriptive statistics for the continuous numeric variables together with the results of the Mann–Whitney-U test are presented in Table 2. Given that the distributions of variables were not normal, median and inter-quartile ranges (IQR) were used as measures of central tendency. The categorical variables are summarized in Table 3. The inflammatory markers including CRP, neutrophil count, monocyte count and RDW were linked to both SCL and in-hospital mortality in univariate statistics. However, NLR showed a link for in-hospital mortality only. Indeed, these results supported our initial idea of applying the above variables to the construction of machine-learned models.

**Table 2:** Basic characteristics of continuous numerical variables grouped by outcomes

	Feature	Unit	Significant lesion			No significant lesion			p-value	In-hospital death			No in—hospital death			p-value
			Support	Median	IQR	Support	Median	IQR		Support	Median	IQR	Support	Median	IQR	
1	Age	Years	4825	65.8	14.3	1778	67	14.8	< *0.001*	94	75.8	18.7	6509	66	14.3	< 0.001
2	Height	cm	4653	170	11	1743	170	11	*0.059*	70	169.5	10.3	6326	170	11	0.303
3	Weight	kg	4697	80	20	1748	80	20	*0.707*	75	78	21	6370	80	20	0.266
4	BMI	kg/m^2^	4643	27.7	6.5	1739	28.1	6.4	*0.044*	70	27	6.3	6312	27.8	6.5	0.173
5	Lymphocyte count	10 e^−3^/ul	4718	1.9	1	1719	1.8	0.9	< *0.001*	84	1.7	1.5	6353	1.9	0.9	0.284
6	Monocyte count	10 e^−3^/ul	4718	0.6	0.3	1719	0.6	0.2	*0.007*	84	0.7	0.5	6353	0.6	0.3	< 0.001
7	Eosinophil count	10 e^−3^/ul	4718	0.1	0.1	1719	0.1	0.1	*0.628*	84	0	0.1	6353	0.1	0.1	< 0.001
8	Neutrophil count	10 e^−3^/ul	4703	5.2	2.9	1707	4.9	2.4	< *0.001*	84	9.1	4.7	6326	5.1	2.8	< 0.001
9	Basophile count	10 e^−3^/ul	4718	0.1	0	1719	0.1	0	*0.426*	84	0.1	0	6353	0.1	0	0.732
10	Haemoglobin level	g/dl	4701	14.4	2.1	1715	14.3	2.1	*0.103*	83	12.8	3.1	6333	14.4	2.1	< 0.001
11	RDW	%	4634	12.2	1.3	1692	12.4	1.3	< *0.001*	84	12.5	1.9	6242	12.2	1.4	0.004
12	Haematocrit	%	4707	42.4	5.9	1715	42.4	5.9	*0.883*	84	38	8.1	6338	42.5	5.9	< 0.001
13	MCV	fL	4718	90.9	6.3	1719	91.5	6.2	< *0.001*	84	92.5	8.6	6353	91	6.2	0.129
14	Platelet count	10 e^−3^/ul	4718	223	79	1719	214	77	< *0.001*	84	236	112	6353	221	78	0.392
15	MPV	fL	4682	8.5	2.1	1688	8.6	2.3	< *0.001*	84	8.4	2.3	6286	8.5	2.2	0.238
16	NLR	Ratio	4703	2.7	2	1707	2.6	1.8	*0.081*	84	5.1	5	6326	2.6	1.9	< 0.001
17	PLR	Ratio	4718	115.3	65.6	1719	115.7	63.3	*0.926*	84	131.1	125.1	6353	115.2	64.4	0.097
18	Fibrinogen	mg/dl	4612	403	128	1715	398	124	*0.357*	83	439	173.5	6244	401	126	0.027
19	LDL	mg/dl	3561	109	66	1465	95	57	< *0.001*	59	99	49.5	4967	104	64	0.236
20	HDL	mg/dl	3588	48	18	1480	51	21	< *0.001*	59	45	16	5009	49	20	0.001
21	Total Cholesterol	mg/dl	3580	177	71	1474	164	65	< *0.001*	59	163	61	4995	173	71	0.026
22	Triglycerides	mg/dl	3560	122	89	1469	117	78	*0.004*	59	109	78.5	4970	121	85	0.352
23	CRP	mg/dl	1067	0.7	3	443	0.4	1.4	< *0.001*	36	5.5	9.4	1474	0.6	2.3	< 0.001
24	TSH	μU/ml	3975	1.3	1.2	1588	1.4	1.3	< *0.001*	69	1.5	1.8	5494	1.3	1.3	0.127
25	Urea	mg/dl	3227	37	17	843	38	18	*0.125*	49	50	33	4021	37	17	< 0.001
26	Creatinine	mg/dl	4712	1	0.4	1727	1	0.4	*0.011*	84	1.3	0.6	6355	1	0.4	<0.001
27	GFR	ml/min	4468	76	29	1684	73	29	< *0.001*	79	52	30.5	6073	75	29	< 0.001
28	Glycated Haemoglobin	%	349	6.4	1.9	69	6.4	1.6	*0.731*	7	7.9	1.8	411	6.3	1.8	0.009
29	Sodium	mmol/l	4766	141	4	1745	141	4	< *0.001*	85	138	6	6426	141	4	< 0.001
30	Potassium	mmol/l	4765	4.4	0.6	1745	4.4	0.5	< *0.001*	84	4.3	0.8	6426	4.4	0.6	0.8
31	Prothrombin time	s	4721	13.3	1.3	1748	13.4	1.5	*0.065*	87	15.2	3.2	6382	13.3	1.4	< 0.001
32	Thrombin time	s	4322	16	1.5	1680	16.1	1.4	< *0.001*	73	16.3	2.5	5929	16	1.4	0.166
33	Heart rate at admission	1/min	4943	72	18	1826	72	18	*0.166*	97	79	30	6672	72	18	0.012
34	Systolic blood pressure	mmHg	4943	120	20	1826	120	20	*0.044*	97	100	40	6672	120	20	< 0.001
35	Diastolic blood pressure	mmHg	4943	80	17	1826	80	12	*0.07*	97	70	18	6672	80	12	< 0.001
36	Troponin I level	ng/l	2446	0	0.8	551	0	0.1	< 0.001	40	6.3	35.4	2957	0	0.6	< 0.001
37	Troponin T level	ng/l	2344	0	0.1	1210	0	0	< 0.001	42	0.5	1.7	3512	0	0.1	< 0.001
38	Alanine transaminase	U/l	2289	24	18	1077	23	16	0.012	47	29	47	3319	24	17	0.006
39	Aspartate transaminase	U/l	2321	24	16	1100	24	11	0.003	48	50	101.2	3373	24	14	< 0.001

The p-values apply to the univariate Mann–Whitney-U test

*IQR* inter-quartile range, *BMI* body mass index, *RDW* red cell distribution width, *MCV* mean cell volume, *MPV* mean platelet volume, *NLR* neutrophil to lymphocyte ratio, *PLR* platelet to lymphocyte ratio, *LDL* low density lipoprotein, *HDL* high-density lipoprotein, *CRP* C-reactive protein, *TSH* thyroid stimulating hormone, *GFR* glomerular filtration rate

**Table 3:** Basic characteristic of nominal features divided by target groups

	Feature	Values	Count where significant lesion; n = 4943 (100%)	Count where no significant lesion; n = 1826 (100%)	Count, where patient died in hospital; n = 97 (100%)	Count where no in-hospital death; n = 6672 (100%)
1	CABG during hospitalisation or planned after discharge	Not qualified	4174 (84%)	1826 (100%)	89 (91.8%)	5991 (90%)
		Qualified	769 (16%)	0	8 (8.2%)	761 (11%)
2	Dysglycemia	No	3489 (71%)	1319 (72%)	63 (64.9%)	4745 (71%)
		Yes	1454 (29%)	507 (28%)	34 (35.1%)	1927 (29%)
3	Cardiac arrest	False	4879 (99%)	1818 (100%)	77 (79.4%)	6620 (99%)
		True	64 (1%)	8 (%)	20 (20.6%)	52 (1%)
4	Hypertension	True	4584 (93%)	1701 (93%)	82 (84.5%)	6203 (93%)
		False	359 (7%)	125 (7%)	15 (15.5%)	469 (7%)
5	PCI during hospitalization	True	4247 (86%)	0	76 (78.4%)	4171 (63%)
		False	696 (14%)	1826 (100%)	21 (21.6%)	2501 (37%)
6	Smoking	Former Smoker	2565 (52%)	1020 (56%)	44 (45.4%)	3541 (53%)
		Non-Smoker	1272 (26%)	528 (29%)	33 (34. %)	1767 (26%)
		Active Smoker	1106 (22%)	278 (15%)	20 (20.6%)	1364 (20%)
7	History of CABG	False	4546 (92%)	1542 (84%)	89 (91.8%)	5999 (90%)
		True	397 (8%)	284 (16%)	8 (8.2%)	673 (10%)
8	History of PCI	False	3366 (68%)	1065 (58%)	80 (82.5%)	4351 (65%)
		True	1577 (32%)	761 (42%)	17 (17.5%)	2321 (35%)
9	History of myocardial infarction	False	3886 (79%)	1358 (74%)	79 (81.4%)	5165 (77%)
		True	1057 (21%)	468 (26%)	18 (18.6%)	1507 (23%)
10	Sex	Male	3342 (68%)	1138 (62%)	56 (57.7%)	4424 (66%)
		Female	1488 (30%)	641 (35%)	38 (39.2%)	2091 (31%)
11	Affected artery	Not specified	619 (13%)	1793 (98%)	18 (18.6%)	2394 (36%)
		RCA	1525 (31%)	8 (< 1%)	23 (23.7%)	1510 (23%)
		LAD	1531 (31%)	2 (< 1%)	37 (38.1%)	1496 (22%)
		Cx	770 (16%)	0 (< 1%)	11 (11.3%)	759 (11%)
		OM	191 (4%)	1 (< 1%)	1 (1%)	191 (3%)
		D	109 (2%)	1 (< 1%)	0	110 (2%)
		LM	108 (2%)	2 (< 1%)	5 (5.2%)	105 (2%)
		Graft	90 (2%)	3 (< 1%)	0	93 (1%)
12	History of heart failure	False	4197 (85%)	1511 (83%)	66 (68%)	5642 (85%)
		True	746 (15%)	315 (17%)	31 (32%)	1030 (15%)
13	History of renal failure	False	4633 (94%)	1679 (92%)	84 (86.6%)	6228 (93%)
		True	310 (6%)	147 (8%)	13 (13.4%)	844 (13%)
14	History of peripheral atherosclerosis	False	4604 (93%)	1674 (92%)	89 (91.8%)	6189 (93%)
		True	339 (7%)	152 (8%)	8 (8.2%)	483 (7%)
15	History of stroke	False	4734 (96%)	1727 (95%)	92 (94.8%)	6369 (95%)
		True	209 (4%)	99 (5%)	5 (5.2%)	303 (5%)
16	Death during hospitalisation	False	4860 (98%)	1812 (99%)	0	6672 (100%)
		True	83 (2%)	14 (1%)	97 (100%)	0

The predictive performance of logistic regression, XGBoost, and the DRSA-BRE classifiers were assessed in a computational experiment. The parameters of all classifiers were based on the training data only. The classification performance was verified in a stratified fivefold cross-validation which was repeated ten times to improve the repeatability of the obtained results. Table 4 provides the summary of their predictive performance.

**Table 4:** Best predictive performance results in fivefold cross-validation of classifiers trained on the simplified set and the full set of features

	Sensitivity [%] (recall)	Specificity [%]	Accuracy [%]	G-mean [%]	AUC	
Logistic regression	78 ± 25	30 ± 31	65 ± 10	48.4^a^	54 ± 3	Significant lesion
Xgboost	*56 *±* 18*	58 ± 20	57 ± 8	57.0^a^	57 ± 2	
DRSA-BRE (full set of features)	56.9 ± 0.2	66.9 ± 0.2	59.6 ± 0.2	61.7 ± 0.02	61.9^a^	
Logistic regression	47 ± 34	90 ± 11	89 ± 10	65.0^a^	68 ± 11	In-hospital death
Xgboost	80 ± 9	79 ± 4	80 ± 4	79.5^a^	78 ± 3	
DRSA-BRE	79.3 ± 1.7	80.6 ± 0.5	81.0 ± 0.5	79.9 ± 1	80.8^a^	
DRSA-BRE (full set of features)	81.0 ± 2.4	81.1 ± 0.5	81.0 ± 0.5	81.0 ± 1	81.0^a^	

^a^Indicates that value was not directly estimated during experiments

The results presented in Table 4 indicate a remarkably better performance of classifiers in detecting in-hospital mortality than SCL. DRSA-BRE and XGBoost trained with the majority class undersampling performed equally well both in the case of in-hospital mortality and SCL. Logistic regression was undoubtedly the worst classifier of all. Considering the characteristics of the compared classifiers, we focused our attention on sensitivity and specificity measures. G-mean was measured during experiments with DRSA-BRE and was calculated afterwards for logistic regression and XGBoost. AUC, by contrast, was measured only for logistic regression and XGBoost and was approximated for DRSA-BRE based on the measured sensitivity and specificity. DRSA-BRE was also able to handle nominal attributes directly [19]. Hence, the experiments with the full set of features were carried out only with DRSA-BRE.

These experiments, nevertheless, indicated that the full set of features did not contribute to a high increase of predictive performance with respect to the simplified set of features. The best result for in-hospital mortality was achieved by DRSA-BRE: 81.03 ± 2.4% sensitivity, and 81.06 ± 0.5% specificity. The best result for SCL was also achieved by DRSA-BRE: 56.91 ± 0.2% sensitivity, and 66.94 ± 0.2% specificity. These results were obtained with the full set of features. When the simplified set of features was used, DRSA-BRE and XGBoost achieved a comparable predictive performance. The comparison of predictive performance measured by G-mean and AUC leads to similar conclusions. Following the obtained results, we focused our further analysis on the detection of in-hospital mortality since the prediction performance of considered classifiers for SCL was not satisfactory.

**Fig. 1:**
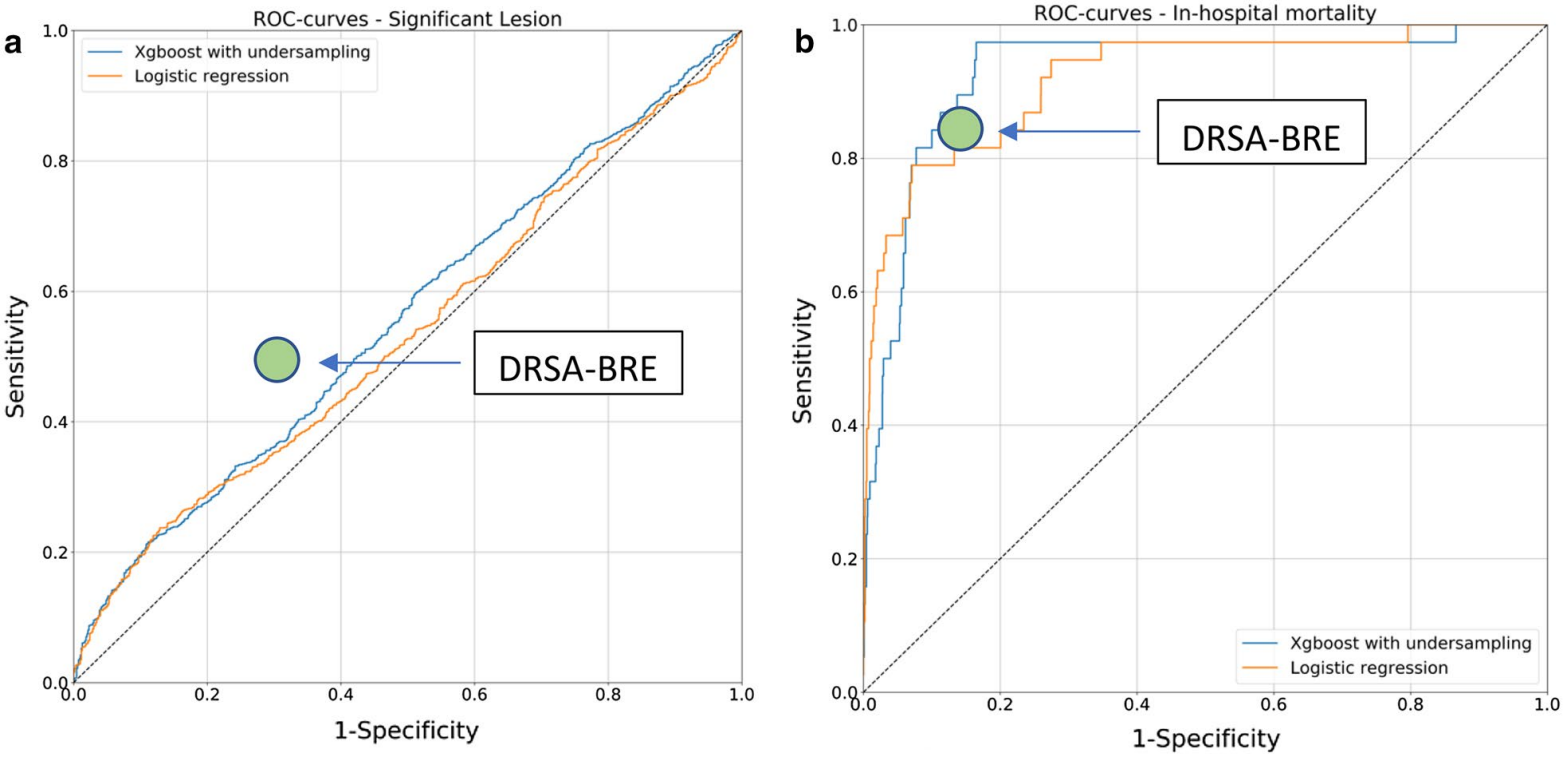
Receiver operating curves presenting the performance of XGboost and logistic regression in the detection of significant coronary lesion and in-hospital death. The green dot represents the approximate performance of the DRSA-BRE classifier

Figure 1a, b presents ROC curves for evaluated classifiers. The Xgboost algorithm was superior in terms of sensitivity while logistic regression achieved higher specificity scores, which can also be observed in the ROC curves. These differences, however, might not be significant, and we concluded that the performance of these classifiers was similar in both classification tasks.

**Fig. 2:**
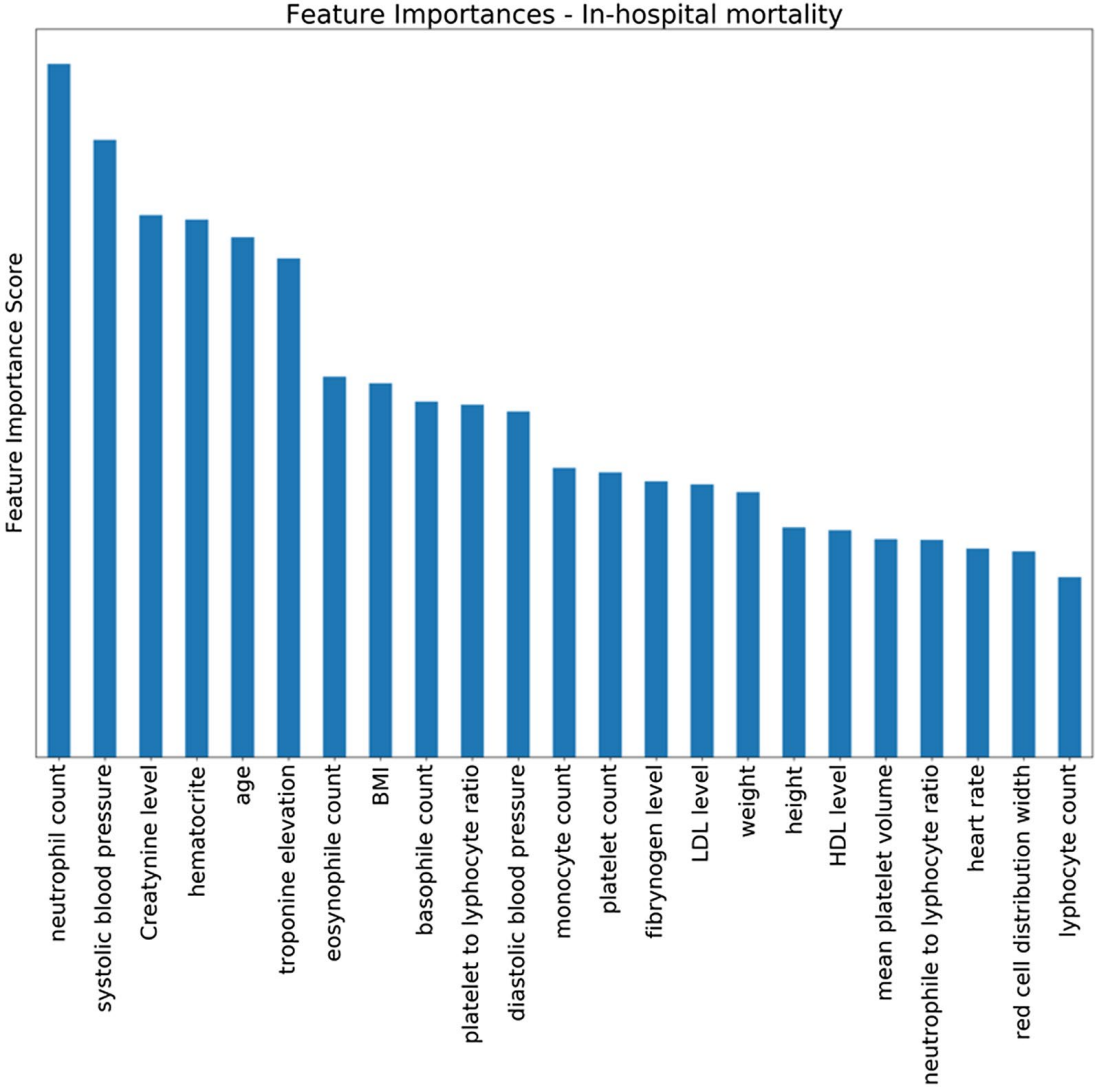
Averaged feature importance scores for the prediction of in-hospital death provided by the XGboost classifier

**Fig. 3:**
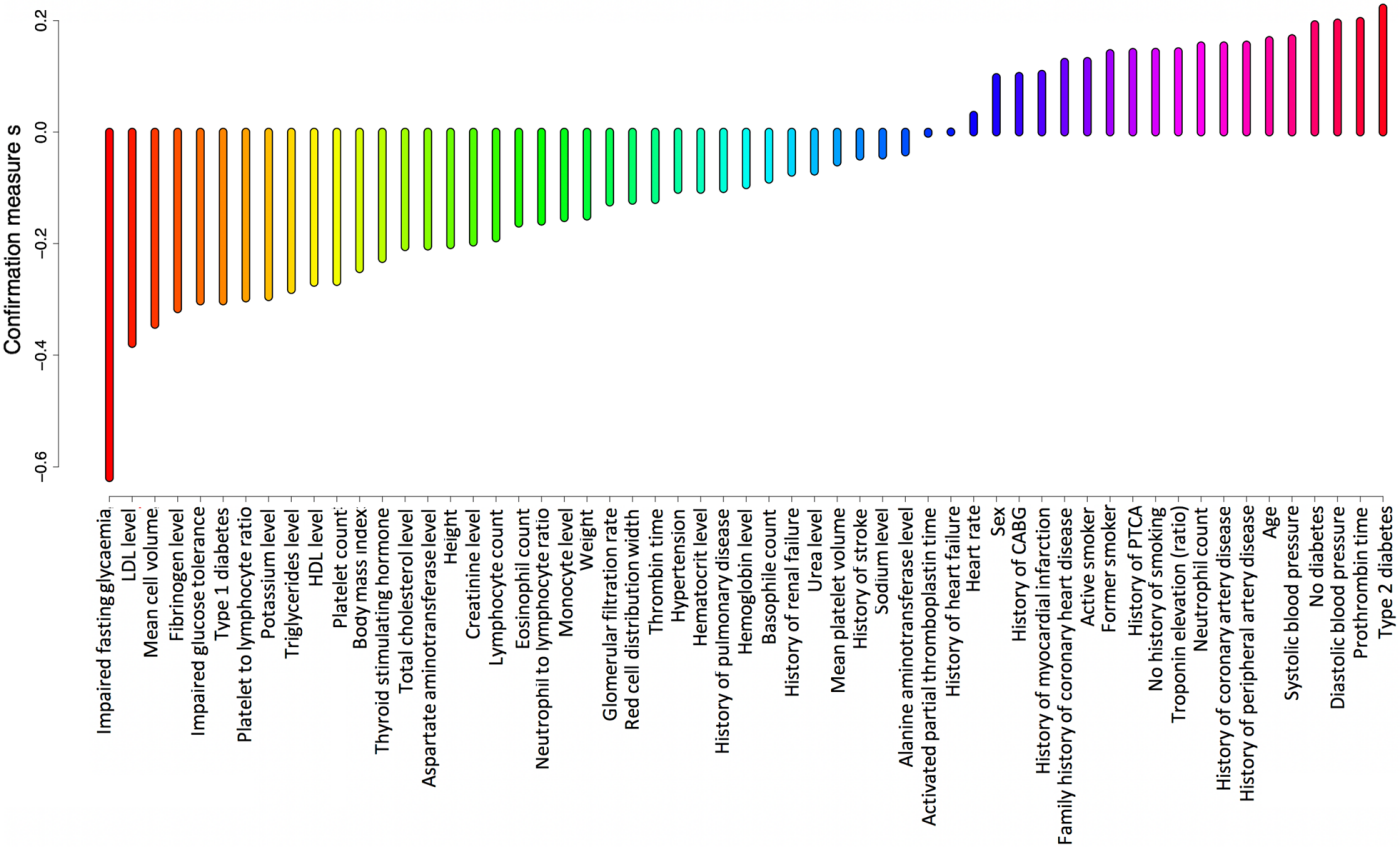
Confirmation measures for the detection of in-hospital death provided by the DRSA-BRE classifier (full set of features)

**Fig. 4:**
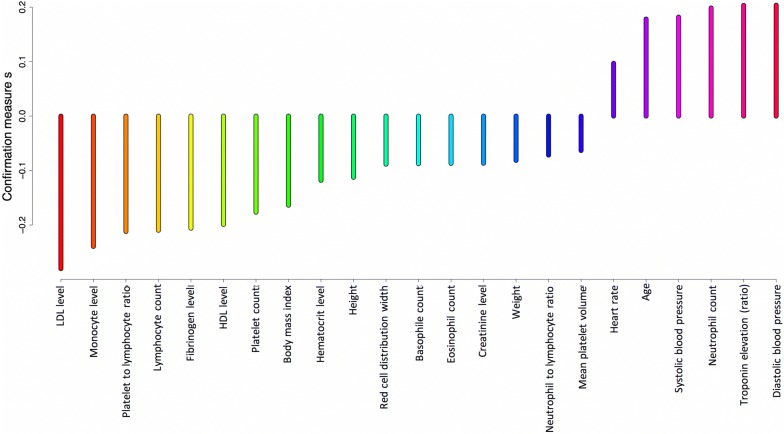
Confirmation measures for the detection of in-hospital death provided by the DRSA-BRE classifier (simplified set of features)

Figure 2 presents relative importance scores for the detection of in-hospital mortality. The top 5 most informative features were: neutrophil count, systolic blood pressure, creatinine level, age and hematocrit. Figures 3 and 4 present confirmation measures provided by the DRSA-BRE classifier (full and simplified set of features, respectively). The features with positive confirmation measures in the simplified set included heart rate, age, diastolic and systolic blood pressure, neutrophil count and troponin elevation. This set partially overlaps with the features of the highest importance provided by the XGboost classifier. The features with positive confirmation measures in the full data set included many clinical features such as diabetes, smoking addiction, previous coronary interventions, MI and peripheral artery disease, which are known to be associated with the outcomes of coronary artery disease. Interestingly, the classifier that used that many features performed only slightly better over the classifier trained on the simplified set (G-mean 81.0 ± 1 vs 79.9 ± 1). As was mentioned above, the simplified algorithms used hematological inflammation markers, the anthropometric data and simple measurements (heart rate and blood pressure).

The analysis of strong decision rules which were induced by DRSA-BRE may allow to investigate the relationship between the features and their values. That effectively may lead to the detection of in-hospital mortality. The selected rules extracted from the DRSA-BRE classifier are presented below.

- Rule 1: If systolic blood pressure ≤ 80 and neutrophil count ≥ 7.14, then in-hospital death occurs;
- Rule 2: If systolic blood pressure ≤ 90 and troponin elevation ratio ≥ 5.29, then in-hospital death occurs;
- Rule 3: If systolic blood pressure ≤ 80 and RDW ≥ 12.7, then in-hospital death occurs;
- Rule 4: If systolic blood pressure ≤ 80 and NLR ≥ 3.06, then in-hospital death occurs.

## Discussion and limitations

Decision rules based on the DRSA-BRE algorithm reflect some well-known mortality risk factors in ACS. It is remarkable that most rules selected by the DRSA-BRE classifier are also present in the Global Registry of Acute Coronary Events (GRACE) risk score. The GRACE risk score has been extensively validated in multiple studies and its use is currently recommended in the guidelines of the European Society of Cardiology [11].

As it is known, low systolic blood pressure may often be related to a cardiogenic shock. Thus, the low value of systolic blood pressure was included in the majority of strong decision rules. What is more, troponin elevation corresponds to the size and severity of the infarction. The neutrophil to lymphocyte ratio and the red cell distribution width are also known to correlate with the ACS outcomes [1, 2, 27]. Interestingly, it was reported that RDW and the mean platelet volume (MPV) combined with the GRACE risk score results improved its predictive value. However, we found no publications on attempts to create a model that relies mostly on laboratory test results.

Numerous studies exploring the application of ML techniques in the diagnostics of ACS focused primarily on risk stratification in patients with chest pain who were admitted to the emergency room (ER). VanHouten et al. [28] applied random forests and elastic net algorithms to a data set of over 20,000 patients admitted to the ER with chest pain. Their results achieved high accuracy with AUC = 0.85, outperforming both the TIMI and GRACE scores. Their much wider selection of patients indicated that 41.9% of them were considered positive for an ACS event. In our study, due to selection bias (patients were already classified by doctors as having a high chance of SCL), it seemed impossible to make a prediction of SCL based on the laboratory test results only, regardless of which classifier was used.

We identified possible causes of the unsatisfactory performance in detecting SCL. The retrospective data analysis made it possible to use a significant amount of data collected in electronic records but also implies many limitations. Patients were selected for the study based on discharge diagnosis which can introduce a selection bias. In our dataset, there were relatively many records with co-morbidities like the history of heart failure (15.6%) or diabetes (29%) as well as with the history of PCI (34%) or CABG (10%). It might be caused by the fact that for patients who were admitted multiple times during the analyzed period, every hospitalization was included in the study dataset.

Troponin levels are known to have high sensitivity and specificity in detecting myocardial ischemia. However, in our study, we were analyzing laboratory results retrospectively and during the analyzed period different type of troponin assays were used. Moreover, the specificity of troponin elevation in the detection of SCL among patients with chronic heart failure is lower. This might have also affected the performance in detecting SCL.

Wallert et al. [29] used a large multi-center register combined with the data from the Swedish national death registry to predict a 2-year survival vs non-survival. They achieved AUC = 0.77 on their data set of over 50,000 patients. The classification was based on 39 predictors. The best performing model was based on linear regression and age was identified as the most predictive factor.

Fonarow et al. developed a useful and straightforward algorithm based on decision trees to predict in-hospital mortality in acutely decompensated heart failure [30]. It identified low admission systolic blood pressure, high admission creatinine and urea nitrogen levels as the best predictors for mortality. Low systolic blood pressure and elevated creatinine are known predictors of short- and long-term mortality in ACS and are used in the GRACE risk score. In our study the analysis of confirmation measures (provided by the DRSA-BRE algorithm) and feature importance scores (provided by XGboost algorithm) confirmed the high predictive value of these features for short-term mortality.

When analyzing the data retrospectively, it is common to have certain values missing. Some laboratory tests are performed under specific conditions only, which in itself may comprise a confounding factor. Moreover, many variables that have been analyzed in this study can be influenced by numerous health conditions. For example, a patient with a high neutrophil count could have suffered from a severe infection which—as a result—may have affected his/her chance of survival. These features might not be specific enough improve detection of SCL but performed well in predicting in-hospital mortality.

## Conclusion

The existing risk scores for the ACS outcomes partially rely on the information from clinical examination. Our results suggest that it may be possible to achieve good outcome predictions on the basis of simple routine measurements that can be obtained without the additional involvement of a physician. This might be of key importance in busy departments where similar systems integrated with electronic medical records could automatically flag high risk patients.

Both DRSA-BRE and the model of gradient boosted trees algorithm for the detection of in-hospital mortality achieved high sensitivity and specificity which makes these models potentially applicable. However, to make a justified statement about the performance of our machine learning models in a clinical setting, they need to be tested prospectively on a different group of patients. Our attempts to detect SCL brought no desired results. This leads to a conclusion that it is not possible to predict the presence of SCL in patients with ACS using the features discussed in this paper.

Inflammatory processes play a key role in the development of atherosclerosis and destabilization of plaques. Our study confirms the findings regarding the important role of neutrophil count in the prognosis of short-term ACS outcomes. However, we could not confirm the prognostic value of the platelet to lymphocyte ratio. The neutrophil to lymphocyte ratio was only associated with in-hospital mortality in univariate tests.

## Abbreviations

ACS: acute coronary syndrome
BMI: body mass index
CABG: coronary artery bypass grafting
IQR: inter-quartile range
MCV: mean cell volume
MPV: mean platelet volume
NLR: neutrophil to lymphocyte ratio
PLR: platelet to lymphocyte ratio
LDL: low-density lipoprotein
HDL: high-density lipoprotein
CRP: C-reactive protein
GFR: glomerular filtration rate
RDW: red cell distribution width
SCL: significant coronary lesion
DRSA-BRE: Dominance-based Rough Set Approach Balanced Rule Ensemble
